# Wenshen Jianpi recipe, a blended traditional Chinese medicine, ameliorates proteinuria and renal injury in a rat model of diabetic nephropathy

**DOI:** 10.1186/s12906-019-2598-1

**Published:** 2019-07-30

**Authors:** Xiaodan Cao, Renxiong Wei, Jun Zhou, Xiaoxia Zhang, Wenbo Gong, Tinglong Jin, Xiabo Chen

**Affiliations:** 1 Department of Clinical Laboratory, Ningbo Municipal Hospital of Traditional Chinese Medicine, Affiliated to Zhejiang Chinese Medical University, Ningbo, 315000 People’s Republic of China; 2 Department of Endocrinology, Ningbo Municipal Hospital of Traditional Chinese Medicine, Affiliated to Zhejiang Chinese Medical University, Ningbo, 315000 People’s Republic of China

**Keywords:** Wenshen Jianpi recipe, Diabetic nephropathy, Podocyte, Nephrin, Podocin

## Abstract

**Background:**

Wenshen Jianpi recipe (WSJPR), a blended traditional Chinese medicine, is considered to have the possible beneficial effect on the progression of diabetic nephropathy (DN). This present study was designed to elucidate this protective activity in a rat model with streptozotocin (STZ)-induced DN and to explore the possible underlying mechanism.

**Methods:**

Adult Sprague Dawley (SD) rats were induced to develop DN through intraperitoneal injection of STZ (60 mg/kg). Animals were orally administered saline, WSJPR at 7.5, 15, 30 g/kg, and valsartan (25 mg/kg) daily for 8 weeks. Blood and 24-h urine samples of each rat were collected for biochemical examination at 2-week intervals. Microcirculatory blood flow in the renal cortex and hemorheology index were also measured. At the end of 8 weeks, all rats were sacrificed to obtain the kidney tissues for histological examination and reverse transcription polymerase chain reaction (RT-PCR) was used to analyze the transcriptional levels of nephrin and podocin genes.

**Results:**

WSJPR could improve serum total protein (TP) and albumin (ALB), reduce the excretion rates of urine-TP (U-TP), urine-ALB (U-ALB) and urine urea nitrogen (UUN) (*P* < 0.05), although it did not significantly alter the hyperglycemia. In addition, treatment with WSJPR could strongly reduce blood flow, erythrocyte aggregation index, and ameliorate microcirculation. In histological measurement, WSJPR-treated rats showed a significant amelioration in glomerular hypertrophy and mesangial expansion. By RT-PCR, we found WSJPR up-regulated the nephrin and podocin expression at mRNA levels.

**Conclusion:**

This study suggested that WSJPR could effectively relieve renal damage and improve renal function of DN rats by ameliorating metabolism disorder and increasing the gene expression of nephrin and podocin, which might be a useful approach for the treatment of DN.

## Background

Diabetic nephropathy (DN) is a major microvascular complication of diabetes and the leading cause of end-stage renal disease (ESRD) in many developed countries [1]. DN is characterized by the increase in the glomerular filtration rate with intraglomerular hypertension and clinically progressive albuminuria followed by eventual loss of renal function [2, 3]. Podocyte is an important component of the glomerular filtration barrier and plays a critical role in renal function injuries in DN [4]. Podocyte damage is mainly manifested as the retraction of their foot processes, detachment, and abnormal expression of key proteins such as nephrin and podocin, which lead to a series of renal structure abnormalities including glomerular basement thickening, mesangial expansion and glomerulosclerosis [5–9]. The general clinical use of multitudinous interventions focused on managing hyperglycemia and high blood pressure do not efficiently lower or reverse the progression of nephropathy, and a considerable proportion of diabetic patients still suffer from progressive and severe renal injury [10–12]. Therefore, there is an urgent need to develop new and effective renoprotective approaches to the treatment of DN.

In China, traditional Chinese medicine (TCM) has been in practice for thousands of years in the treatment of diabetes and its complications [13], showing a number of remarkable results and becoming more popular worldwide [14, 15]. Wenshen Jianpi recipe (WSJPR) is a TCM preparation developed from famous and experienced TCM doctors, which consists of seven herbs (*Aconiti Lateralis Radix Praeparata*, *Zingiber officinale Roscoe*, *Radix Codonopsis*, Rhizoma *Atractylodis macrocephalae*, *Poria cocos*, Radix *Paeoniae Alba*, *Glycyrrhiza uralensis*)*.* In our earlier investigation, the therapeutic effects of four kinds of TCM on streptozotocin (STZ) induced DN rats were observed and WSJPR has shown significant effect on decreasing proteinuria. However, the therapeutic mechanisms of this herbal combination are not clear.

In the current investigation, the STZ induced DN rat model was used to evaluate the effects of WSJPR on renal function and renal pathological changes and the potential causal mechanisms.

## Methods

### Drugs

WSJPR is composed of seven raw materials listed in Table 1 [16] and the dried herbs were provided by Ningbo Municipal Hospital of Traditional Chinese Medicine, Zhejiang, China. The herbal materials were morphologically identified by Dr. Wenbo Gong. Voucher specimen of each species was deposited at Ningbo Municipal Hospital of Traditional Chinese Medicine. The crude drugs were boiled with distilled water for 5 h and filtered. The filtered liquid was condensed to a concentration of approximately 3 g/ml, stored at 4 °C and diluted with distilled water before use [16].

**Table 1:** The composition of WSJPR

Ingredient	Weight
*Aconitum lycoctonum*	6-11 g
*Zingiber officinale*	6 g
*Codonopsis pilosula*	15 g
*Atractylodes macrocephala*	15 g
*Wolfiporia extensa*	15 g
*Paeonia lactiflora*	15 g
*Glycyrrhiza uralensis*	3 g

### Animals

Male, 6-week-old healthy Sprague Dawley (SD) rats (240 g–280 g) were purchased from Shanghai Experimental Animal Centre, Chinese Academy of Sciences. All animals were housed in a specific-pathogen-free facility with ad libitum access to food and water under a constant temperature (23 ± 1 °C) and a 12-h light/dark cycle. This study was carried out in accordance with the recommendations of the Guidelines for the Care and Use of Laboratory Animals of the Ministry of Science and Technology of China. The protocol was approved by the Institutional Animal Care and Use Committee at Zhejiang Chinese Medical University Laboratory Animal Research Center (Zhejiang, China) (Approval Number: IACUC-20190225-05).

### Experimental procedures

Streptozotocin (STZ, Sigma, USA) was used to induce diabetes as described previously [17–19]. All the 58 rats were limited to water access for 12 h before the experiments. 50 rats were administered by a single intraperitoneal injection of 60 mg/kg body weight of STZ dissolved in citrate buffer (0.1 mol/L, pH 4.5). On day 15, the fasting blood glucose (FBG) levels of diabetic animals were measured and those with persistent hyperglycemia over 16.7 mmol/L were selected as diabetes. 40 randomly selected diabetic rats were then divided into five groups: STZ control; STZ + WSJPR 7.5 g/kg; STZ + WSJPR 15 g/kg; STZ + WSJPR 30 g/kg; STZ + valsartan 25 mg/kg (positive control group). The remaining eight rats received one dose intraperitoneal injection of citrate buffer only were used as normal control group. The rats were then administrated saline 10 mL/kg (normal and STZ controls), three different concentration of WSJPR or valsartan 25 mg/kg by an oral gavage method once a day for 8 weeks. Body weight, urinary volume, and biochemical parameters were monitored every 2 weeks. The rats were anaesthetized by intraperitoneal injection of 3% pentobarbital sodium (45 mg/kg) for the measurements of microcirculatory blood flow in the renal cortex. At the end of the experiments, rats were sacrificed by cervical dislocation and kidneys were excised and kept in liquid nitrogen before tests.

### Biochemical analysis

Body weight of rats was measured at one week intervals. Blood was sampled at 2-week intervals for measurement of FBG, blood urea nitrogen (BUN), serum creatinine (Cr), total protein (TP) and albumin (ALB). The measurements of these indexes were conducted by using standard biochemical kits (Jiancheng, Institute of Biotechnology, China), respectively. 24-h urine of each rat was collected individually by metabolic cage at 2-week intervals. Susequently, the urine volume, urine-glucose (U-GLU), urine-UN (UUN), urine-TP (U-TP) and urine-ALB (U-ALB) were tested and the excretion rate was assessed as: concentration * urine volume/24 h.

### Measurements of blood flow and Hemorheology index

The microcirculatory blood flow measurements were performed in the renal cortex in each animal by laser doppler flowmeter (Moor, UK) and it was expressed as ml·min^− 1^·g^− 1^ [20]. The blood samples were obtained from heart and heparin anticoagulant tubes were used as the sampling tubes. The systematical changes of hemorheology of all rats were investigated by SA-5600 automatic hemorheology detector (Succeeder, China) [21].

### Renal histological analysis

Kidney sections were fixed in 4% buffered paraformaldehyde, embedded in paraffin, sectioned at 4 μm thickness and stained with hematoxylin and eosin (HE) and periodic acid schiff (PAS) to assess the alterations of glomerular. The sections were examined with light microscopy (Olympus, Japan) and micrographs were obtained randomly with magnification of 400 × .

### Reverse transcriptase polymerase chain reaction (RT-PCR)

Total RNA was extracted from the kidney tissue of SD rats with Trizol Reagent (Invitrogen, USA), quantitated by measuring the OD260 and OD280 ratio, and then digested with gDNA Eraser (Takara, China) to remove genomic DNA. RNA was reverse-transcribed using oligo-dT random primers, with the superscript II reverse transcriptase (Invitrogen, USA), following the manufacturer’s instructions. Aliquots of cDNA were amplified by PCR using primers specific for rat nephrin, podocin and a constitutively expressed housekeeping gene, β-actin. Primers for PCR were designed by primer 5.0 according to the Genebank sequence and synthesized by Sangon Biotech Co., Ltd. (Shanghai, China). PCR reactions were performed in 7500 Real-Time PCR system (Applied Biosystems, USA) using the following conditions: 95 °C for 30 s followed by 40 cycles of 95 °C for 5 s, 60 °C for 34 s, 72 °C for 40 s, and a final extension step at 72 °C for 10 min. The quantity of specific mRNA was normalized to the expression level of internal control β-actin mRNA.

### Statistical analysis

Data were expressed as mean ± SD. Statistical comparisons were performed with one-way analysis of variance by GraphPad Prism 5.0. Differences with *P* value < 0.05 were considered statistically significant.

## Results

### General observations and symptoms

**Fig. 1:**
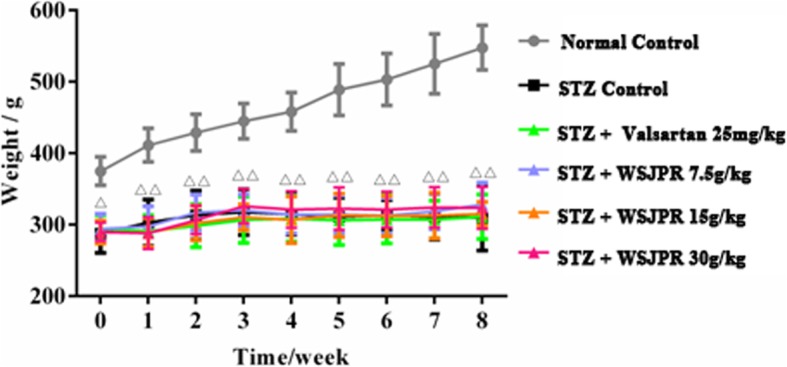
Effect of WSJPR on body weight in rats at different times (*n* = 8). ^*△*^*P* < 0.05, ^*△△*^*P* < 0.01 vs. normal control; mean ± SD

During the 8-week experiment period, rats in the normal control group exhibited no apparent fluctuations in behavior or physiological appearance and obtained progressive gain of body weight. However, the rats in the STZ control group displayed depression, body weight loss, reduced activity, and a dull coat, all of which were typical manifestations of DN. The body weights of rats in the STZ control group were significantly lower (*P* < 0.01) than those in the normal control rats (Fig. 1), which persisted for the duration of the study. The body weights of rats in the WSJPR and valsartan treatment groups increased to varied extent compared to the STZ control group, but there was no significant difference. The mean weight of the positive control group rats was lower than that of rats in the WSJPR treatment group (Fig. 1).

### Relevant biochemical variables

**Fig. 2:**
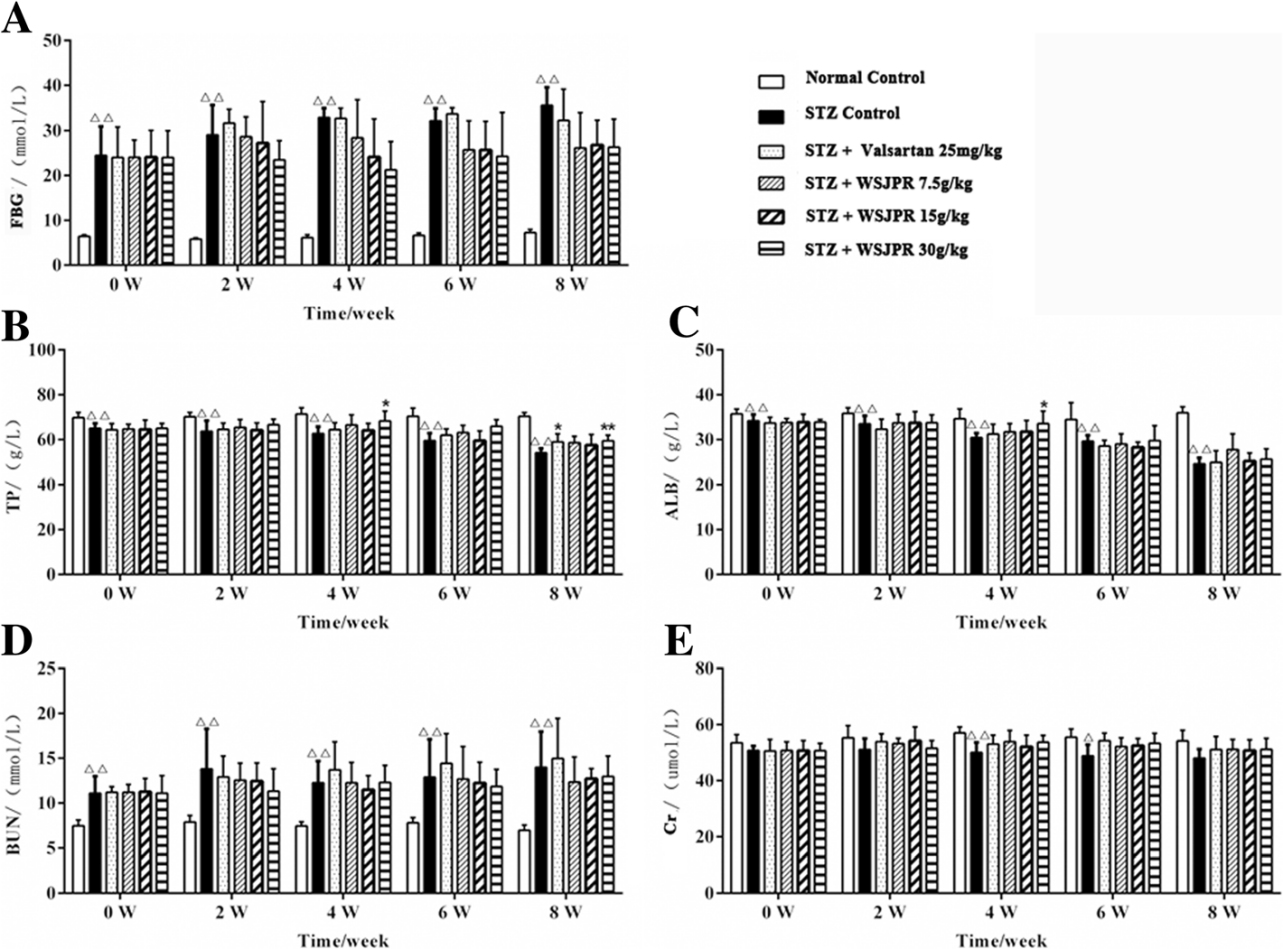
Effect of WSJPR on FBG (**a**), serum TP (**b**), ALB (**c**), BUN (**d**), and Cr (**e**) in rats at different times (*n* = 8). ^*△*^*P* < 0.05, ^*△△*^*P* < 0.01 vs. normal control and ^*^P < 0.05, ^**^P < 0.01 vs. STZ control; mean ± SD

**Fig. 3:**
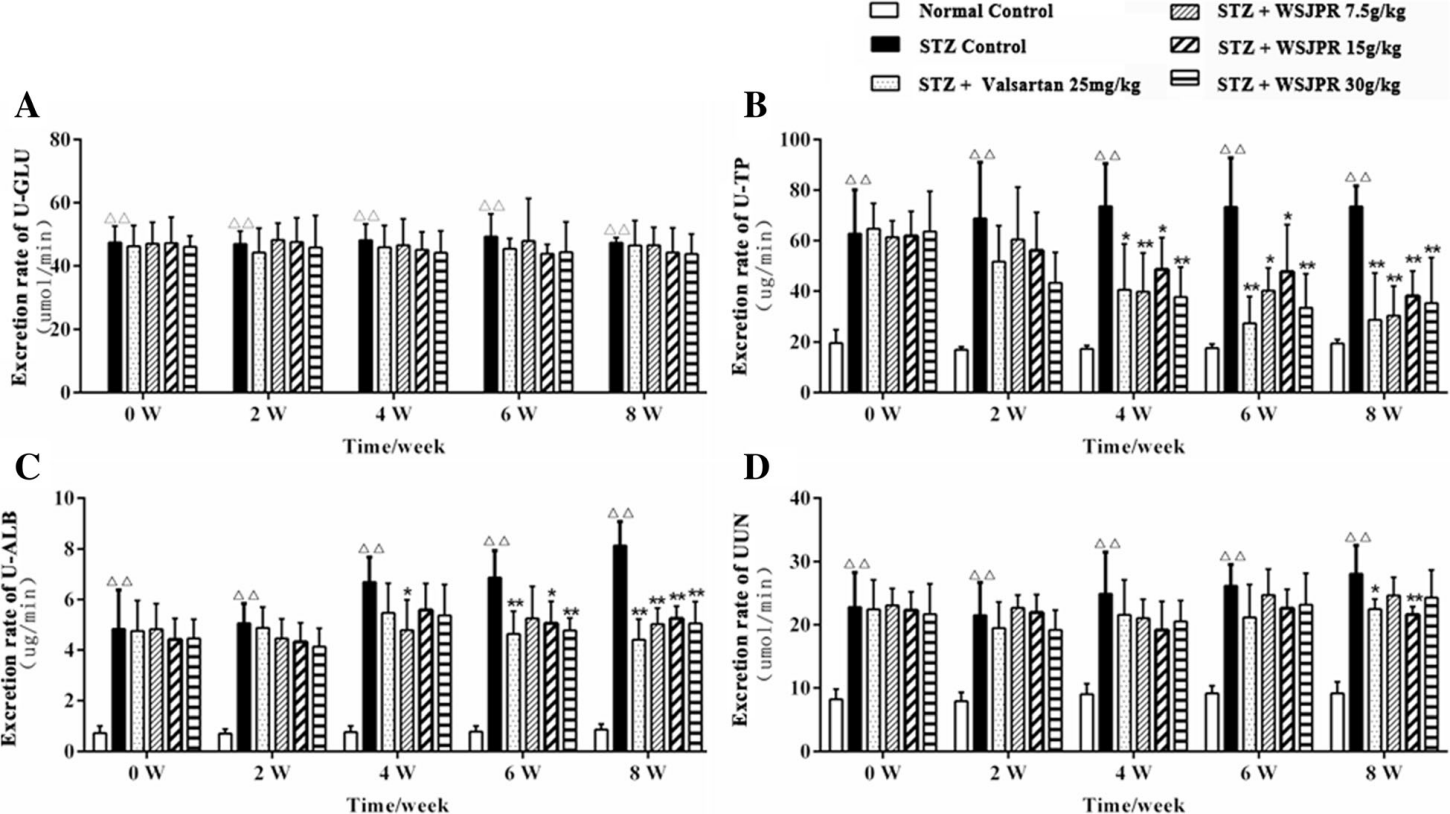
Effect of WSJPR on the excretion rate of U-GLU (**a**), U-TP (**b**), U-ALB (**c**) and UUN (**d**) in rats at different times (*n* = 8). ^*△*^*P* < 0.05, ^*△△*^*P* < 0.01 vs. normal control and ^*^P < 0.05,^**^P < 0.01 vs. STZ control; mean ± SD

The FBG and BUN levels were significantly higher while the TP and ALB levels were significantly lower in STZ control rats than those in normal control (*P* < 0.01) (Fig. 2a-d). The changes in the levels of TP and ALB were significantly reversed by WSJPR (30 g/kg) at week 4 or 8 (Fig. 2b, c). Significant differences (*P* < 0.05) were found in serum concentrations of Cr between the normal control and STZ control group at week 4 and 6 (Fig. 2e). The FBG, BUN and Cr levels in the WSJPR and valsartan treatment groups did not reach statistical significance in comparison to the STZ control group (Fig. 2a, d, e). The excretion rate of U-GLU, U-TP, U-ALB and UUN were all significantly elevated in STZ control group compared to normal control during the 8-week study period (*P* < 0.01). WSJPR and valsartan treatments markedly attenuated the excretion rate of U-TP, U-ALB and UUN (*P* < 0.05) and they did not obviously affect the U-GLU excretion rate in DN rats (*P* > 0.05) (Fig. 3a-e).

### Microcirculatory blood flow analysis

**Fig. 4:**
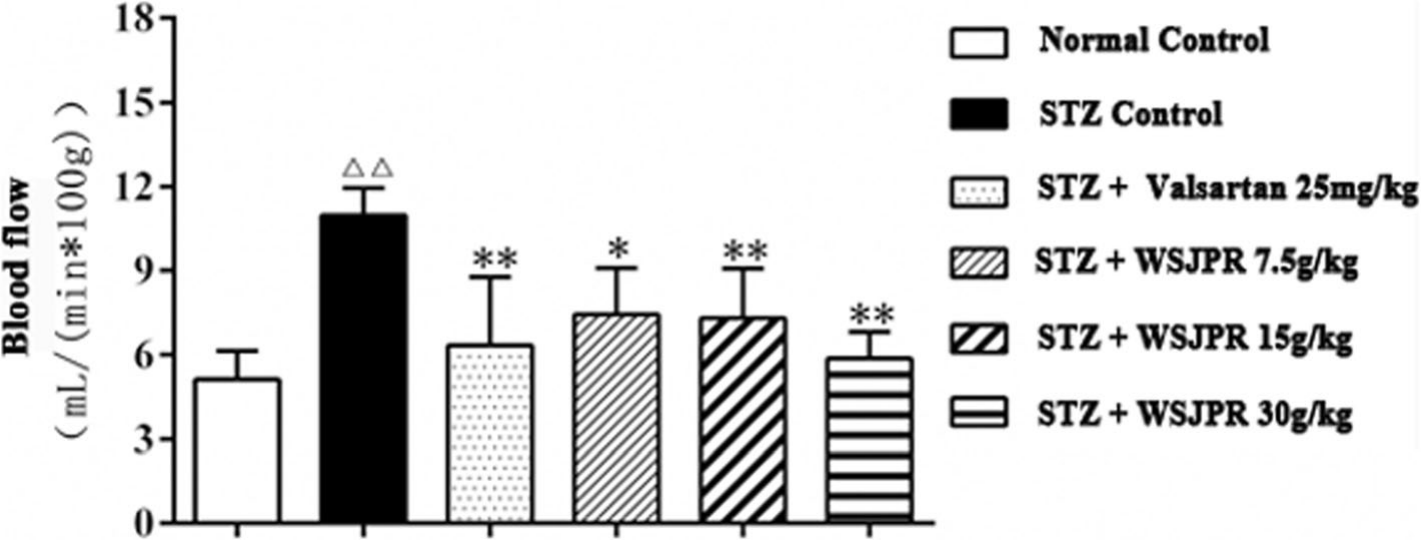
Effect of WSJPR on the microcirculation blood flow in rats renal cortex (*n* = 8). ^*△△*^*P* < 0.01 vs. normal control and ^*^P < 0.05, ^**^P < 0.01 vs. STZ control; mean ± SD

The microcirculatory blood flow of renal cortex was significantly higher in STZ control rats than in normal control rats (*P* < 0.01). The results showed that the blood flow was obviously decreased after treatment with WSJPR and the effect appeared to be dose dependent (Fig. 4). In addition, the blood flow was also markedly decreased after treatment with valsartan (*P* < 0.01).

### Hemorheology indexes analysis

As shown in Table 2, compared with the normal control group, the erythrocyte count, hematocrit and erythrocyte aggregation index in the STZ control group were significantly increased (*P* < 0.05), while the erythrocyte deformation index showed a downward trend (*P* > 0.05). Compared with the STZ control group, erythrocyte count, hematocrit and erythrocyte aggregation index displayed a downward trend (*P* > 0.05), and erythrocyte deformation index exhibited an upward trend (*P* > 0.05) in the WSJPR and valsartan treatment groups. The differences of erythrocyte aggregation index among the treatment groups (WSJPR 30 g/kg, valsartan 25 mg/kg) and STZ control group were statistically significant (*P* < 0.01).

**Table 2:** Hemorheology indexes analysis in rats of each group (mean ± SD, *n* = 8)

Group	Normal control	STZ control	STZ + WSJPR 7.5 g/kg	STZ + WSJPR 15 g/kg	STZ + WSJPR 30 g/kg	STZ + valsartan 25 mg/kg
Hematocrit (L/L)	0.49 ± 0.03	0.55 ± 0.03^△△^	0.52 ± 0.02	0.52 ± 0.02	0.52 ± 0.01	0.52 ± 0.03
Erythrocyte aggregation index	8.14 ± 0.98	11.19 ± 2.21^△△^	10.53 ± 1.32	9.94 ± 1.83	7.97 ± 1.57^**^	5.51 ± 1.81^**^
Erythrocyte deformation index	0.95 ± 0.09	0.86 ± 0.06	0.91 ± 0.06	0.93 ± 0.08	0.88 ± 0.09	0.71 ± 0.16
Erythrocyte rigidity index	7.70 ± 1.31	7.30 ± 1.63	7.66 ± 1.22	8.31 ± 1.86	7.02 ± 1.96	6.97 ± 1.62
Erythrocyte count (× 10^12^/L)	5.29 ± 0.32	5.89 ± 0.34^△^	5.62 ± 0.19	5.62 ± 0.18	5.56 ± 0.15	5.63 ± 0.28
Erythrocyte electrophoresis time(S)	19.24 ± 2.06	18.18 ± 0.9	17.71 ± 2.42	18.28 ± 2.87	16.96 ± 0.95	14.84 ± 3.74

^△^*P* < 0.05 and ^△△^*P* < 0.01 vs. normal control group, ^**^P < 0.01 vs. STZ control group

### Renal histopathology

**Fig. 5:**
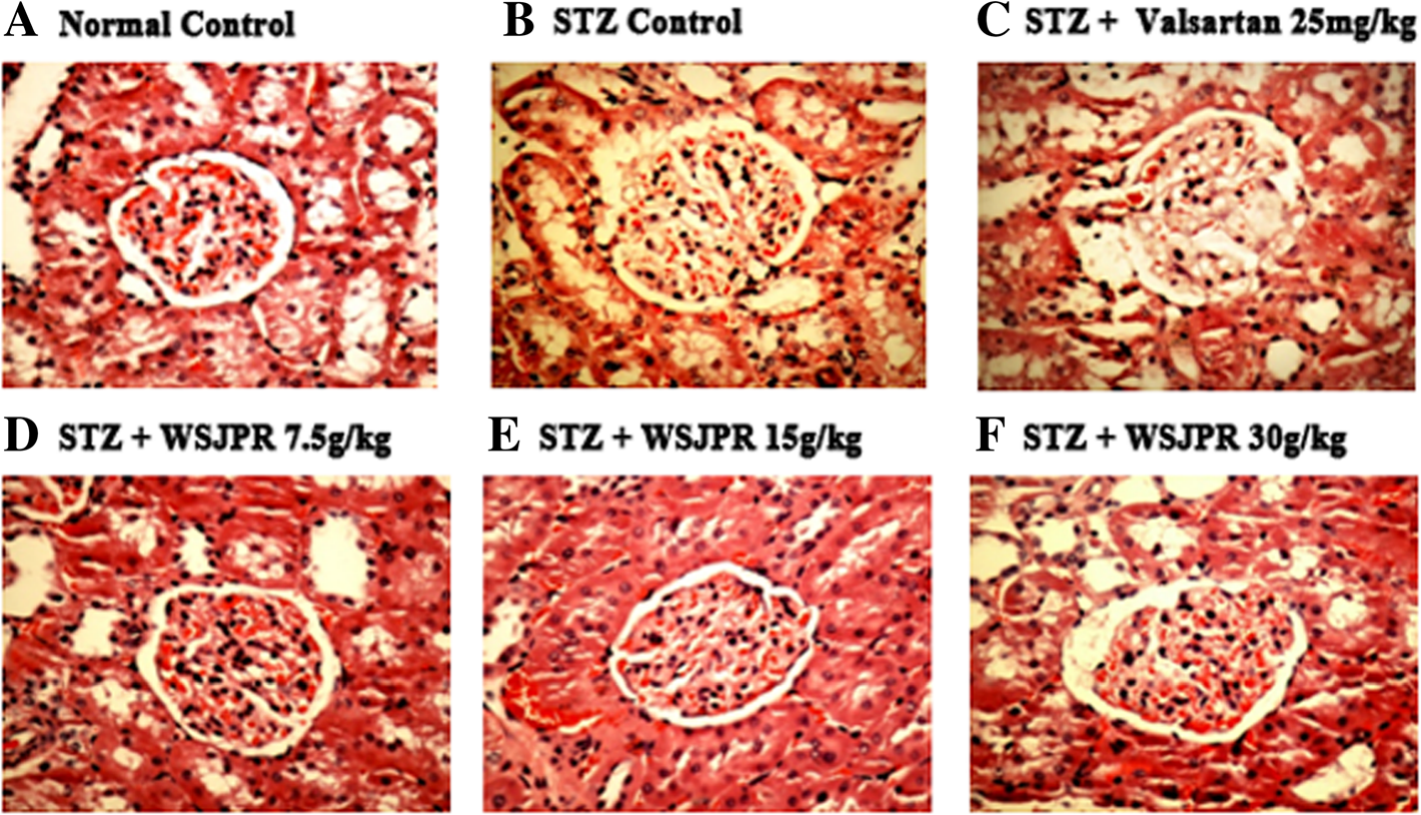
Effect of WSJPR on the renal histomorphology in rats (HE staining, 400× magnification). **a** Normal control; **b** STZ control; **c** STZ + valsartan 25 mg/kg; **d** STZ + WSJPR 7.5 g/kg; **e** STZ + WSJPR 15 g/kg; **f** STZ + WSJPR 30 g/kg

**Fig. 6:**
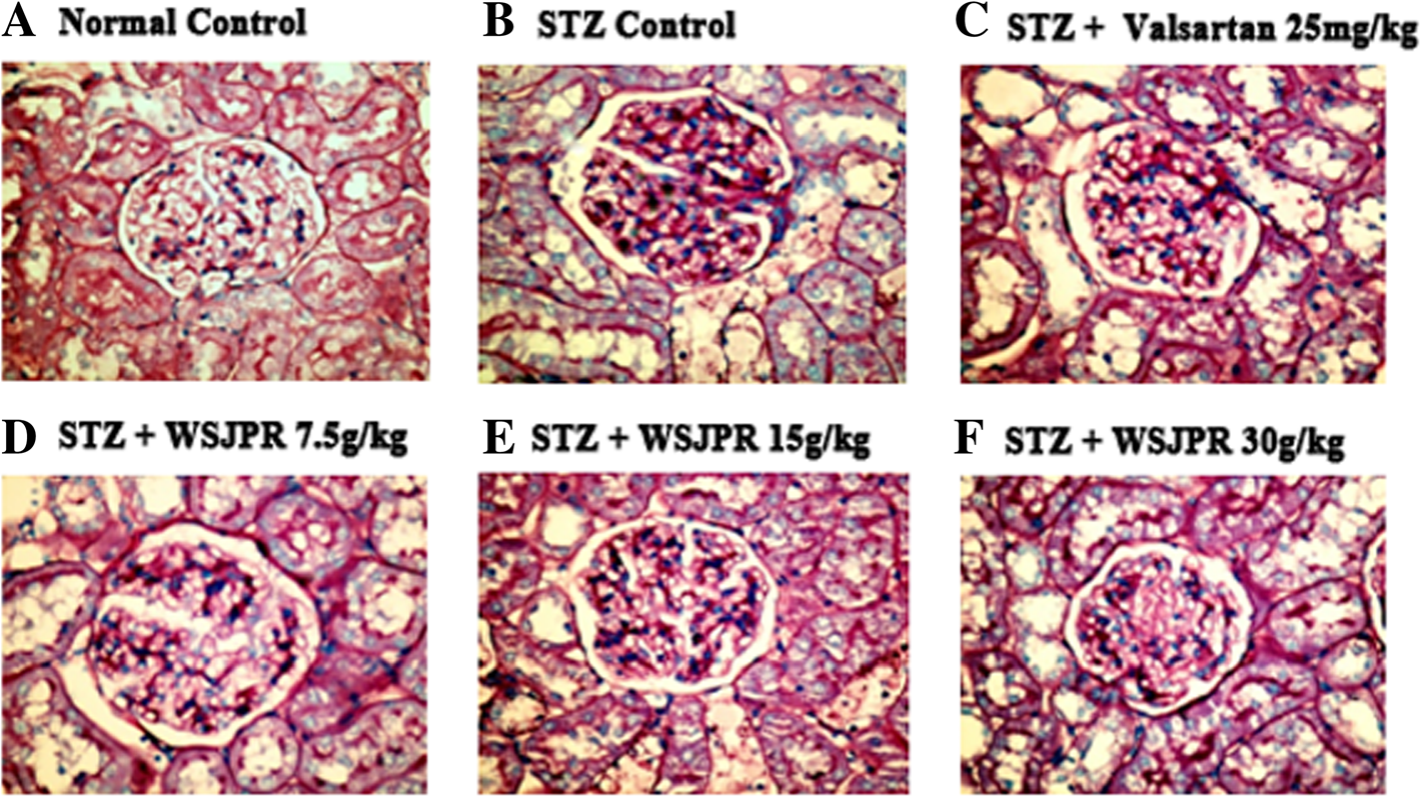
Effect of WSJPR on the renal histomorphology in rats (PAS staining, 400× magnification). **a** Normal control; **b** STZ control; **c** STZ + valsartan 25 mg/kg; **d** STZ + WSJPR 7.5 g/kg; **e** STZ + WSJPR 15 g/kg; **f** STZ + WSJPR 30 g/kg

Glomerular structures were examined by HE and PAS staining respectively. The STZ control rats had notable glomerular hypertrophy, mesangial matrix expansion and basement membrane thickening (Fig. 5a, b). After eight weeks treatment with WSJPR, those histopathological alterations of glomerular structure induced by STZ were significantly inhibited (Fig. 5d-f). PAS staining further validated the renal histological improvements in WSJPR treatment groups and demonstrated that WSJPR had an inhibition of DN-induced glycogen collection (Fig. 6]a, b, d-f). In addition, treatment with valsartan ameliorated these changes to some degree compared with that of STZ control rats (Figs. 5c and 6c).

### mRNA expression of Nephrin and Podocin in renal tissues

**Fig. 7:**
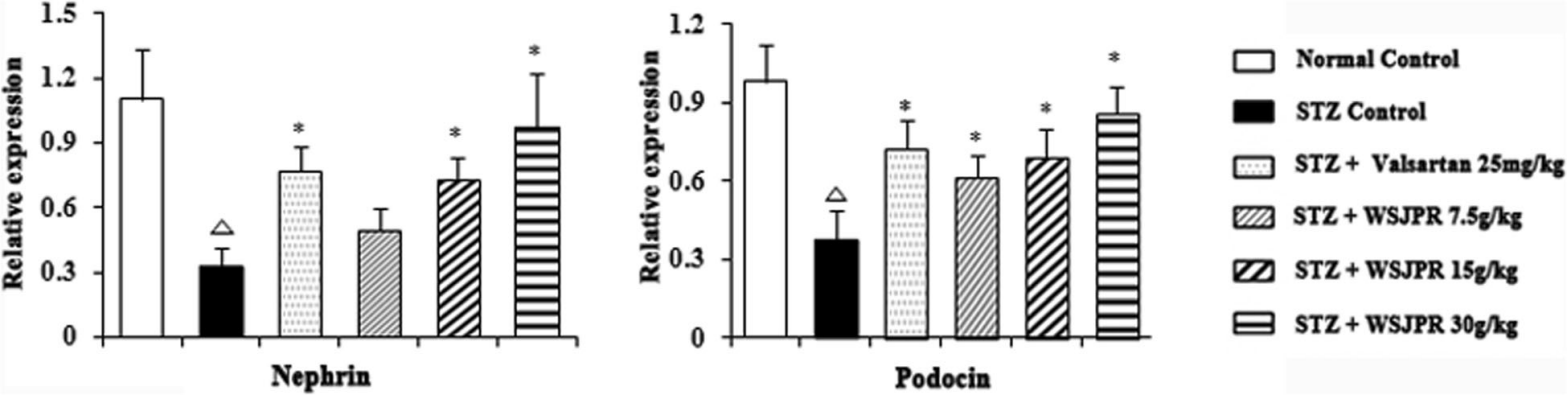
Effect of WSJPR on the transcriptional levels of nephrin and podocin gene in rats (*n* = 8). ^*△*^*P* < 0.05 vs. normal control and ^*^P < 0.05 vs. STZ control; mean ± SD

RT-PCR revealed that significant reductions in the relative amounts of nephrin and podocin mRNA were apparent in STZ control rats compared to relative levels in the normal control rats (*P* < 0.05). WSJPR remarkably increased the mRNA expression level of nephrin and podocin in the renal tissues dose dependently, compared with the STZ control group (*P* < 0.05). Treatment with valsartan injection significantly modulated the level of nephrin and podocin in DN rats (*P* < 0.05) (Fig. 7) and their expression levels were more profound with WSJPR (30 g/kg) than in the valsartan group.

## Discussion

The treatment of diabetes and its complications usually focus on insulin, angiotensin-converting enzyme inhibitor and angiotensin receptor blocker due to their approved efficacy in clinical and animal researches [22, 23]. But these methods are also connected with side effects such as hypovolemia and high proportion of DN patients still progress to ESRD and require everlasting and costly dialysis [24, 25]. Traditional herbal compounds offer a significant advantage and utility for the management of DN via diverse ways and are recognized as effective alternatives to conventional medicine [26, 27]. The present research investigated the effect of a Chinese herbal formulation on structure and function in the kidney of DN rats and valsartan was used as positive control.

In the current study, we used the diabetic rats induced by STZ injection to examine the efficacy and therapeutic mechanisms of WSJPR in DN. The results indicated that FBG, BUN, and the excretion rate of U-GLU, U-TP, U-ALB, UUN were significantly increased and body weight, serum TP, ALB, Cr were significantly decreased in STZ-induced DN rats, and mesangial matrix expansion was observed in the glomeruli by HE and PAS staining. These data demonstrated the successful induction of DN. In the WSJPR treatment group, WSJPR efficiently attenuated diabetic renal injury via improvement of the serum TP and ALB, reduction of the excretion rate of U-TP, U-ALB, UUN and alleviation of mesangial matrix expansion and glomerular basement thickening. The results indicated that WSJPR could effectively improve renal function and ameliorate protein metabolism disorder in DN rats.

Hemodynamic and hemorheological changes are closely related to the progression of nephropathy. In the early stage of DN, renal hemodynamics were abnormal with high filtration, high perfusion and high internal pressure, which are important causes of urinary protein leakage and glomerular sclerosis [28]. Measurement of blood flow in renal cortex can reflect the state of renal peripheral blood circulation and blood perfusion. Normally, the interlobar artery, arcuate artery and interlobular artery are successively decreased in renal cortical blood flow. The closer to the cortex endings, the lower the blood flow [29]. In our study, the microcirculatory blood flow of renal cortex was significantly increased in STZ control group, which suggested that DN rats showed hemodynamic abnormalities with high glomerular filtration and perfusion. Treatment with WSJPR could strongly reduce blood flow and ameliorate microcirculation.

Hemorheological changes such as hypercoagulability, hyperviscosity and slow blood flow could accelerate renal impairment [21]. This study showed that hematocrit, erythrocyte aggregation index and erythrocyte count were significantly increased in DN rats. Hematocrit is closely associated with whole blood viscosity. The elevated level of erythrocytre aggregation can make erythrocyte easily aggregate in a string and cause elevation of blood viscosity [30]. Treatment with WSJPR (30 g/kg) could markedly decrease erythrocyte aggregation index and the pharmacological mechanism might be connected with the reduction of blood viscosity.

Interestingly, the significant effect of WSJPR on hyperglycemia was not observed in diabetic rats. A study has shown that protecting podocytes from hyperglycemia with podocyte-specific deletion of glucose transporter member 4 can prevent the progression of glomerular disease and diabetes-associated albuminuria independently of glucose uptake [31]. The underlying mechanism of the effect of WSJPR on DN observed in the present study is, however, still unclear and needs further study. These results suggested that WSJPR had potential for use in the protection against DN with prevention of albuminuria and amelioration of renal function independent of blood glucose lowering pathways.

Nephrin and podocin are podocyte-associated proteins and have been proven to be an integral membrane protein of the slit diaphragm. Nephrin, expressed on lateral aspect extending into the slit diaphragm, regulates the cytoskeletal architecture and affects the shape and viability of podocytes, while podocin colocalizes and interacts with cytosolic tail of nephrin in the lipid rafts of the podocyte foot process cell membrane [32–35]. In the present study, results showed that the down regulated expressions of renal nephrin and podocin mRNA were observed in STZ control rat compared to normal control and their transcriptional levels were increased significantly after the 8-week period treatment with WSJPR, which is in accordance with the protein expression trends of nephrin and podocin in STZ control and WSJPR treatment groups in our previous immunohistochemistry assay. These results suggested that the increased mRNA and protein levels of nephrin and podocin in WSJPR treatment group might be a main contributor reducing podocyte injury in STZ-induced DN rat. Furthermore, glomerular hypertrophy and hyperglycemia were also detrimental to podocytes [36]. WSJPR treatment can attenuate glomerular hypertrophy and improve hyperglycemia, resulting in multiple protective effects against podocyte injury.

The present study has limitations as follows. First, the present work focused only on the critical associated proteins of the podocyte and did not further evaluate the signaling pathways involved in repairing podocyte injuries such as the mammalian target of rapamycin (mTOR) signaling pathway, which has been proven to be related to pathological damages in podocyte [37]. Second, the level and effect of autophagy in podocyte injuries was not studied in the present work. Autophagy is a bulk degradation process involved in cytosol recycling and the disassembly of superfluous or damaged organelles that plays a key role in the repair of injured podocytes and contributes to maintaining podocyte function [38]. Insufficient autophagy was involved in the pathogenesis of podocyte loss and significant proteinuria in DN [39]. More studies should investigate the effects of WSJPR on mTOR/autophagy to explore the further therapeutic mechanism for DN.

## Conclusion

In summary, we have demonstrated herein that administration of WSJPR ameliorated STZ-induced proteinuria, metabolic disorders, and microcirculation, attenuated GBM thickness, and therefore postponed the progression of DN through restoring podocyte associated molecular nephrin and podocin expressions. WSJPR could be adopted in clinical settings as a part of alternative medicine for DN patients and more extensive investigation of their mechanisms and evidence-based knowledge is still required.

## Data Availability

The datasets analyzed during the current study are available from the corresponding author on reasonable request.

## Abbreviations

ALB: Albumin
BUN: Blood urea nitrogen
Cr: Creatinine
DN: Diabetic nephropathy
ESRD: End-stage renal disease
FBG: Fasting blood glucose
GBM: Glomerular basement membrane
HE: Hematoxylin and eosin
mTOR: mammalian target of rapamycin
PAS: Periodic acid schiff
RT-PCR: Reverse transcriptase-polymerase chain reaction
STZ: Streptozotocin
TCM: Traditional Chinese medicine
TP: Total protein
U-ALB: Urine-albumin
U-GLU: Urine-glucose
U-TP: Urine-total protein
UUN: Urine urea nitrogen
WSJPR: Wenshen Jianpi recipe
